# Ethical tensions created by AI in the mental health sector

**DOI:** 10.1192/j.eurpsy.2025.244

**Published:** 2025-08-26

**Authors:** J. Wise

**Affiliations:** CNWL NHS Foundation Trust, London, United Kingdom

## Abstract

**Abstract:**

Artifical Intelligecne is sold as a magic solution, and yet its inscrutability poses a major problem. Who is the source of the knowledge? Who is accountable? Who is responsible? Who can edit or train it? Has it been trained on copyrighted material? If so, have the owners been compensated? If not, is it culturally relevant? Has the **‘**black box’ aspect been addressed? Whether a provider of knowledge, a clinical decison aid or a decsion maker, troubling issues arise that as yet have not been solved by legisaltion or the market. We shall explore potential benefits, solutions, and actual problems with real world cases that shed light on where AI may take us, and where we may need constarints that lie beyond medical ethics.

**Disclosure of Interest:**

None Declared

